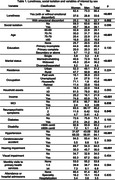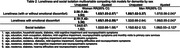# Loneliness, social isolation in older adults and their association with incident dementia in men and women: 3 years of follow‐up on population‐based study

**DOI:** 10.1002/alz.093408

**Published:** 2025-01-09

**Authors:** Brenda Sarai Miranda Sánchez, Isaac Acosta‐Castillo, Ana Luisa Sosa

**Affiliations:** ^1^ Dementia Laboratoy, Instituto Nacional de Neurología y Neurocirugía “Manuel Velasco Suárez”, Ciudad de México, DF Mexico; ^2^ Dementias Laboratory, National Institute of Neurology and Neurosurgery, Mexico City, DF Mexico

## Abstract

**Background:**

Dementia is a syndrome highly prevalent in elderly. Genetic and health factors have been reported to be associated with their onset. There is evidence that some psychosocial factors may have a differential effect by sex, beyond biological or hormonal explanations, as loneliness and social isolation(SI). Loneliness is defined as the feeling of being alone and can be reported as persistent, unavoidable, unpleasant or bothersome. SI is understood as limited or no contact with family or social networks, as well as low social contact, which may include friendly, religious or recreational activities

**Objective:**

to analyze the association between loneliness and social isolation with the risk of incident dementia in older adults, men and women from a population‐based sample.

**Method:**

The analytical sample include 1,517 participants aged ≥65 years evaluated within Dementia Research Group 10/66 in Mexico, between 2006‐2010, from urban and rural areas. Loneliness was assessed based on three items: admits to feeling lonely, with or without emotional discomfort. SI was identified by: friends, relatives and neighbors contacts; living alone and social gatherings attendance. All participants had information for incident dementia, sex, age, education, marital status, residence, occupation, household assets, income, mild cognitive impairment(MCI), neuropsychiatric symptoms(NPS), diabetes, disability, hypertension, cerebral vascular accident(CVA), hearing and visual difficulties, falls and use of health services.

**Result:**

Total prevalence of loneliness and SI were 33.6% and 39.1%, respectively. Women were lonelier, older, not married, without own income, with more hypertension and NPS, than men (see table 1).

In multivariable competing risk models (with death and disability as competing events), loneliness with or without emotional discomfort (RR = 1.63 95% CI:1.03‐2.57) and with emotional discomfort (RR = 1.65 95% CI:1.05‐2.59) showed to be a risk factor for incident dementia in women. Social isolation did not show this effect as well any model for men (see table 2).

**Conclusion:**

Loneliness was shown as a risk factor only in women, effect that was maintained with and without emotional discomfort. We believe that expanding the study of psychosocial factors as well as their usefulness in predicting incident dementia is important, also considering that some of these factors play a different role between men and women.